# Asymmetric multi-band reflective metasurface for linear and circular polarizations conversion in Ku, K, Ka, and U bands

**DOI:** 10.1038/s41598-024-81388-w

**Published:** 2025-02-10

**Authors:** Jamal Zafar, Humayun Zubair Khan, Abdul Jabbar, Jalil ur Rehman Kazim, Masood Ur Rehman, Adil Masood Siddiqui, Qammer H. Abbasi, Muhammad Ali Imran

**Affiliations:** 1 University of Glasgow, Glasgow, G12 8QQ UK; 2 National University of Sciences and Technology, Islamabad, 44000 Pakistan; 3 Artificial Intelligence Research Centre, Ajman University, Ajman, UAE

**Keywords:** Electrical and electronic engineering, Metamaterials

## Abstract

This work proposes a novel multi-band reflective metasurface, that is capable of linear polarization (LP), and circular polarization (CP) conversion in Ku, K, Ka, and U Bands. The metasurface design involves a combination of ring and square elements strategically arranged, and printed on a 0.76 mm thin-grounded Rogers RO3003 substrate. The metasurface achieves LP for *y*-polarized incident electromagnetic (EM) wave in 16.2–17.2 GHz, 23.0–25.4 GHz, 40.3–54.35 GHz frequency bands. The polarization conversion ratio (PCR) for LP frequency ranges is minimum 90% with an fractional bandwidth (FB) of 2.94%, 9.91%, and 26.9%, respectively. Moreover, metasurface achieves CP for *y*-polarized incident EM wave in 16.1–16.55 GHz, 17.5–22.15 GHz, 26.65–37.75 GHz, and 55.6–59.8 GHz frequency bands. In addition, the axial ratio (AR) for CP frequency ranges is less than 3 dB with a FB of 2.75%, 23.45%, 34.47%, and 7.27%. The device performance is considerably stable under oblique incidences up to 45 degrees. The metasurface unitcell is compact with a structural size of $\text {length}=0.23\lambda _\circ$, $\text {width}=0.23\lambda _\circ$ and $\text {thickness}=0.08\lambda _\circ$. The proposed prototype is fabricated, and the measured results are in good agreement with the simulated one. Overall, the proposed metasurface exhibits promising performance characteristics and holds potential for multiple applications in satellite based networks.

## **Introduction**

Linear polarization (LP) and circular polarization (CP) conversion capable metasurfaces operating across the Ku, K, and Ka bands represent a transformative leap in satellite communication (Satcom) technology, revolutionizing the way we transmit and receive data over vast distances. These metasurfaces offer a multitude of benefits that are crucial for optimizing communication systems across these frequency bands^1–3^. The U band is vital for future Satcom due to its potential to alleviate congestion in scarce frequency spectra. Offering increased bandwidth and data capacity, it enables the deployment of advanced communication services, supporting the ever-growing demand for high-speed connectivity and ensuring efficient spectrum utilization in satellite networks^4^.

In the realm of Satcom, efficient spectrum utilization is paramount, and LP metasurfaces play a pivotal role in achieving this goal. By manipulating the polarization state of electromagnetic (EM) waves, these metasurfaces enable the precise control of signal propagation, leading to improved signal-to-noise ratios and enhanced data throughput^5–7^. In the Ku, K, and Ka bands, where a myriad of communication services are deployed, ranging from broadband internet to satellite television broadcasting, the ability to efficiently utilize available bandwidth is critical for meeting the ever-growing demand for high-speed connectivity. Furthermore, CP metasurfaces offer additional advantages in terms of signal resilience and robustness^8^. By imparting CP to transmitted signals, these metasurfaces mitigate the effects of polarization mismatch, atmospheric attenuation, and signal fading, thus ensuring reliable communication links even in adverse environmental conditions. This resilience is particularly crucial for future Satcom systems operating in the U band, where atmospheric attenuation poses a significant challenge to signal integrity^9–11^.

Metasurfaces capable of LP and CP conversion in the Ku, K, Ka, and U bands hold immense potential for present, and future diverse applications in Satcom systems, unlocking new capabilities and enhancing performance across various frequency ranges^12^. In the Ku band, which is extensively utilized for satellite television broadcasting and broadband internet services, these metasurfaces play a crucial role in optimizing signal propagation and mitigating interference, thereby ensuring reliable and high-quality communication links^13^. Additionally, in the K band, where Satcom systems are employed for weather monitoring, Earth observation, and military applications, polarization metasurfaces offer enhanced signal resilience against atmospheric attenuation and environmental factors, ensuring robust communication in challenging conditions^14^.

**Fig. 1:**
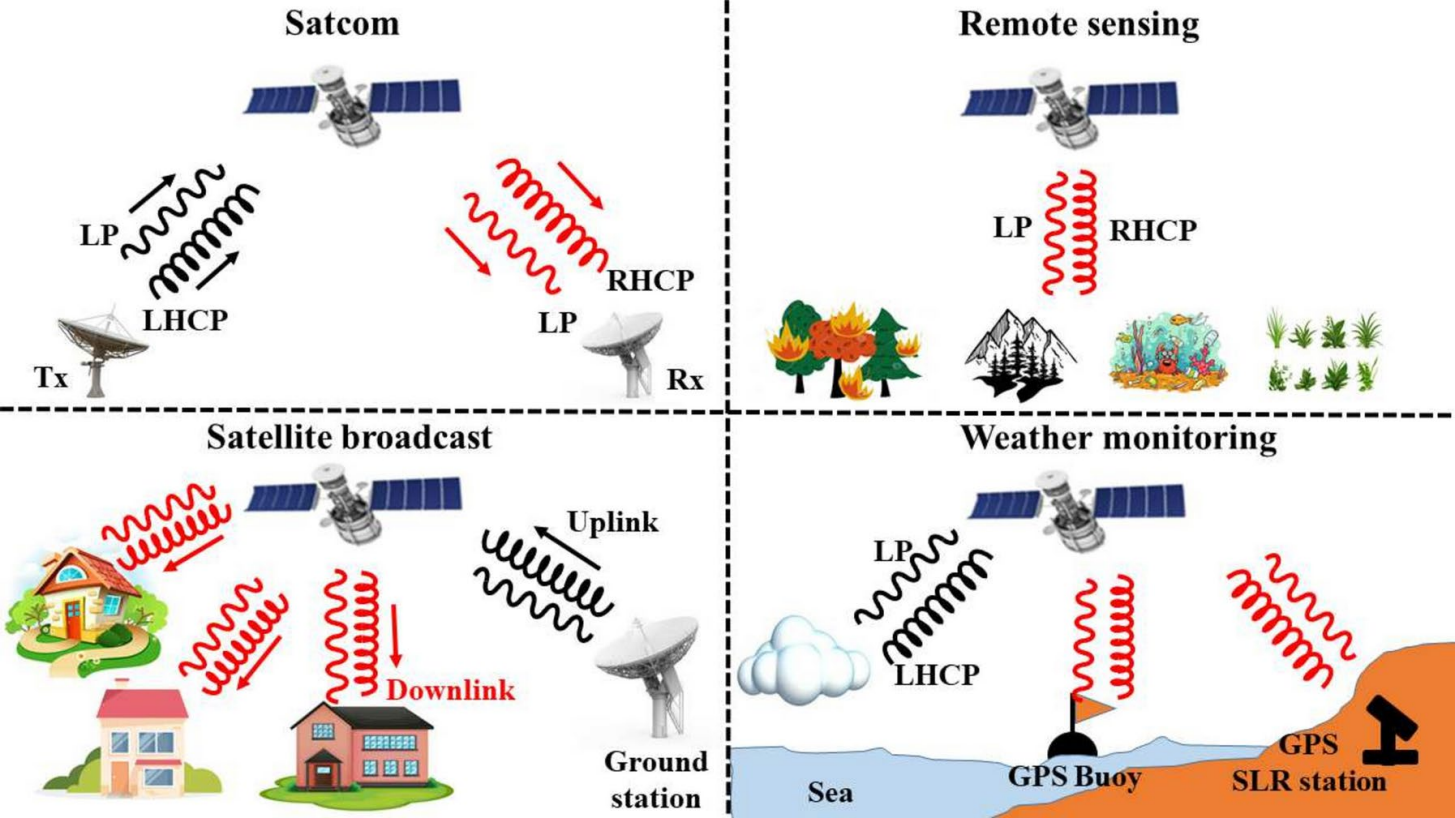
The application scenario for linear and circular polarization.

Moreover, in the Ka band, which is increasingly utilized for high-speed satellite internet services and broadband communication networks, these metasurfaces enable the deployment of advanced antenna designs with adaptive beamforming and electronic steering capabilities, facilitating efficient spectrum utilization and maximizing data throughput^15–17^. Furthermore, in the U band, which holds promise for future Satcom systems due to its potential for increased bandwidth and improved signal quality, LP and CP metasurfaces offer innovative solutions for alleviating congestion in scarce frequency spectra, enabling the deployment of next-generation satellite networks with enhanced capacity and performance^18–20^.

Across all these frequency bands, the application of LP, and CP metasurfaces extends beyond traditional communication services, encompassing a wide range of emerging technologies and applications. From Satcom and remote sensing platforms to satellite-based weather monitoring and satellite broadcast infrastructure, these metasurfaces play a pivotal role in driving innovation and enabling new capabilities in Satellite based networks at the forefront of global connectivity and advancing the frontiers of technology for the benefit of society. Figure 1 shows the application scenarios for LP, and CP in satellite based networks.

The growing demand for high-performance Satcom and radar systems in Ku, K, Ka, and U bands highlights the need for metasurfaces capable of both LP, and CP conversion. These frequency bands are crucial for various satellite applications, including data transmission, remote sensing, and military communications. Traditional devices face challenges in efficiently converting LP and CP across wide frequency ranges, often resulting in limited bandwidth and inconsistent performance. A metasurface capable of multi-band LP and CP conversion offers enhanced flexibility, improved signal quality, and efficient polarization control, meeting the evolving needs of modern communication systems.

### **Literature review**

The existing literature on polarization conversion metasurfaces has expanded significantly, addressing challenges in Satcom and radar applications across Ku, K, Ka, and U bands. This review explores recent advancements, focusing on the development of metasurfaces for efficient LP, and CP conversion, and identifying existing gaps and limitations in past work.

The work in^21^ presents a compact, asymmetric multi-band reflective polarization converter metasurface. The unit cell has a length, and thickness 6 mm, and 2 mm, respectively. It efficiently converts linearly polarized EM waves to their orthogonal counterparts and circularly polarized waves at two frequency ranges in Ku-band. The design, comprising a square with curves and a Split Ring Resonator, achieves successful LP, left hand circular polarization (LHCP), right hand circular polarization (RHCP), verified through simulation and measurement, demonstrating wide angular stability (AS) and high efficiency. The metasurface performs narrow band LP, and CP conversion in Ku band only. The study in^22^ introduces an ultra-thin reflective polarization converter utilizing metasurfaces. The unit cell has a length, and thickness 6.6 mm, and 1.2 mm, respectively. It acts as a hybrid converter, offering both LP, and LHCP while operating in the Ku frequency band, and achieves over 90% polarization conversion ratio (PCR) under normal and oblique angles up to 45 degrees. The metasurface performs multi-band LP, and narrowband CP conversion in Ku band only. The work in^23^ introduces a metasurface-based polarization converter, performing LP, and LHCP in X-, Ku-, and K-band frequencies. The unit cell has a length, and thickness 5.94 mm, and 1.6 mm, respectively. The converter operates efficiently up to 50 degrees oblique incidence EM waves with 80% PCR. The metasurface performs multi-band LP in Ku band, and multi-band CP conversion in Ka band only. The research in^24^ presents a multi-functional metasurface for LP, and CP conversion. The unit cell has a length, and thickness 5 mm, and 3 mm, respectively. Operating in C-, X-, Ku-, and K-bands, it efficiently converts linearly polarized waves to circular and vice versa across multiple frequency ranges with stability under oblique incidence up to 75 degrees, making it versatile for various applications. The metasurface performs narrowband LP conversion in C, and X band, and multi-band CP conversion in Ku, and K band.

The study in^25^ addresses challenges in polarizer design for communication systems, focusing on wide bandwidth, simple design, and stable performance under oblique incidence. The unit cell has a length, and thickness 5.1 mm, and 0.127 mm, respectively. It introduces a polarizer design utilizing split circular ring resonators and square rings, converting linearly polarized waves to LHCP or RHCP in Ku- and Ka-bands. The metasurface performs multi-band CP conversion in Ku, and K band only. The work in^26^ proposes a dual-band miniaturized LHCP converter metasurface with multi-band and wide-angle axial ratio (AR). It consists of periodic unit cells with three metallic layers. The unit cell has a length, and thickness 2.8 mm, and 1.01 mm, respectively. The design achieves multi-band response in K-, and Ka-bands, maintaining an AR below 3 dB over specified bandwidths with AS up to 45 degrees. The research in^27^ proposes a miniaturized multi-band LP, LHCP, and RHCP metasurface-enabled reflective polarizer. The unit cell has a length, and thickness 2.52 mm, and 1 mm, respectively. Utilizing a Meander-line structure, it converts *y*-polarized waves to cross, and circular polarization across multiple frequency bands with stable performance under oblique incidences in K- and Ka-bands. The metasurface performs narrowband LP , and CP conversion in C and X band, and multi-band LP, and CP conversion in Ku, and K band.The study in^28^ analyzes and designs a metasurface-based reflective linear-to-circular polarization converter in planar and conformal profiles. The unit cell has a length, and thickness 4.6 mm, and 0.51 mm, respectively. It utilizes a split circular metallic ring on a metal-backed dielectric substrate, achieving LHCP, and RHCP conversion with AR $\le$ 3 dB at two frequency ranges in Ka-, and U-band maintaining AS up to 25 degrees. The metasurface performs narrowband CP conversion in Ka band, and multi-band CP conversion in U band.

The existing work^21–28^ on metasurfaces for polarization conversion in the Ku, K, Ka, and U bands has encountered several unresolved challenges, particularly regarding bandwidth limitations, reduced PCR, and inconsistent performance across broad frequency ranges. Moreover, the design of the unit cell is not simple, and the size is large. Many designs focused on narrowband solutions, which limited their effectiveness in applications requiring multi-band polarization conversion. Additionally, achieving both LP, and CP conversion across multiple frequency bands simultaneously proved difficult due to complex structural requirements. Material limitations also contributed to poor efficiency and restricted operating frequencies.

Based on the existing literature, it is evident that designing a metasurface with compact dimensions and a simple unit cell structure remains a challenge. Despite various advancements, achieving stable performance under oblique incident EM waves while enabling both LP, and CP conversion across Ku, K, Ka, and U bands is an ongoing area of research. Many previous designs have shown limitations, either in their complexity, large size, or decreased effectiveness under different angles of incidence. Therefore, there remains a significant opportunity for researchers to develop metasurfaces that address these issues with improved efficiency and functionality across multiple frequency bands.

### **Motivation and contribution**

In Satcom applications, it is vital for antennas to not only support CP but also function across two distinct, non-adjacent frequency bands with orthogonal polarizations, i.e., LHCP, and RHCP. This ensures improved signal isolation, especially in environments with very low power flux densities vulnerable to real-time interference. Additionally, effective performance of polarizer metasurfaces at greater oblique angles is crucial due to the unpredictable incidence angles of EM waves. This capability is increasingly critical at higher frequency ranges where extended path lengths of EM waves within a dielectric spacer can lead to destructive interference^29–31^. This study addresses these challenges by proposing a reflective metasurface inspired by prior research^21–28^, aiming to achieve the following objectives while ensuring optimal performance across diverse operational scenarios and drawing insights from recent advancements in the field:

- The metasurface design showcased in this study achieves LP conversion across multiple frequency bands. Specifically, it demonstrates bandwidths of 0.50 GHz in the Ku-band, 2.40 GHz in the K-band, and 14.05 GHz in the U-band, with resonance frequencies at 16.95 GHz, 24.1 GHz, 43.95 GHz, and 52.1 GHz, respectively. Notably, the design achieves remarkable efficiency, boasting a PCR exceeding 90% and significant AS up to 45 degrees.
- The proposed metasurface demonstrates CP across four distinct frequency bands. These bands include a bandwidth of 0.45 GHz in the Ku-band for RHCP, 4.65 GHz in the Ku- and K-band for LHCP, 11.1 GHz in the Ka-band for RHCP, and 4.2 GHz in the U-band for RHCP. Notably, the metasurface maintains a minimum band gap of 0.9 GHz between adjacent bands. Furthermore, it ensures stable AR and ellipticity even at oblique incidences of up to 45 degrees.
- To verify the effectiveness of the proposed design, we fabricated a metasurface that comprises a grid of $100\times 140$ unit cells and conducted experimental comparisons with simulation results. The observed agreement between experimental and simulated polarization conversion outcomes was satisfactory, confirming the validity and reliability of the design’s performance.
- This metasurface design demonstrates its versatility for applications in Satcom, remote sensing, weather monitoring, and satellite broadcast. It is particularly suitable for scenarios where achieving CP (with orthogonality) and LP conversion is paramount.
- The proposed metasurfaces effectively address the unresolved challenges of previous work, such as bandwidth limitations, low PCR, and inconsistent performance across multiple frequency bands. By integrating multi-resonant unit cells and advanced geometric configurations, the design achieves a multi-band polarization conversion spanning the Ku, K, Ka, and U bands. This ensures high PCR across these ranges, overcoming the narrowband limitations of earlier designs. Additionally, the structure’s optimized material composition enhances efficiency, allowing for both LP, and CP conversion. These innovations provide a robust solution for satellite communication applications, ensuring consistent performance across diverse frequency bands.

The paper is structured as follows: Section II presents design and theory, section III gives the simulation and analysis. In Section IV, we present design, parametric study, surface current distribution in detail. Section V presents fabrication and experimental measurements. Finally, Section VI concludes this paper.

## **Design and theory**

### **Unit cell design**

A standard unit cell for polarization-converting metasurfaces features a layered structure: a patterned copper top layer, a dielectric middle, and a metallic base. The top metallic design intricately interacts with incoming electromagnetic waves, altering their polarization. The dielectric layer offers mechanical stability and adjusts wave velocity, influencing resonance frequencies. The bottom metallic layer serves as a reflector, redirecting waves back through the metasurface, ensuring effective polarization conversion, such as linear (LP) and circular (CP) polarization. This combination of materials and structure is key for efficient wave manipulation across various frequency bands.

**Fig. 2:**
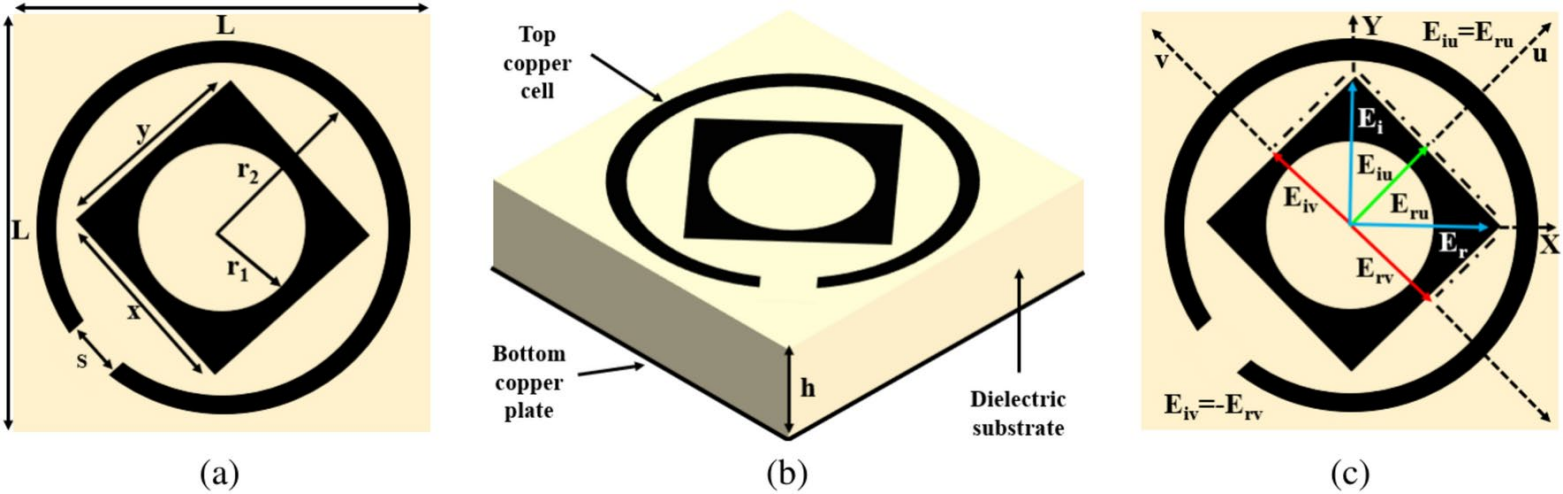
The unit cell (**a**) Front view (**b**) Perspective view (**c**) UV diagram.

The proposed metasurface’s structural design is illustrated in Fig. 2, showcasing a unit cell of the asymmetric reflective metasurface. The design process encompasses three key components: a top copper cell, an intermediate dielectric substrate, and a bottom copper plate. When an incident EM wave interacts with the structure, it generates *x*- and *y*-polarized transmitted and reflected EM waves. Through multiple reflections between these transmitted waves and the bottom copper plate, the ultimate reflected wave emerges. Notably, the phase and magnitude of the reflected EM wave can be intentionally controlled by regulating interactions within the dielectric and ground plane. To ensure the structure remains compact, careful selection of parameters is crucial, particularly for the thickness of the dielectric spacer where multiple reflections occur. The top and bottom layers are separated by an Rogers RO3003 substrate with a permittivity of $\epsilon _r=3.00 \pm 0.04$ and a loss tangent of $\delta =0.0010$, with a thickness of $h=0.76$ mm. The top and bottom layers feature copper with electrical conductivity $\sigma =5.8 \times 10^7$ S/m and thickness $t=0.035$ mm.

The design procedure commences with a square-shaped unit cell of dimension $L \times L \hspace{2pt} \text {mm}^{2}$. Then, a square of dimensions $x \times y$ $\text {mm}^{2}$ rotated at $45^\circ$ with a cylindrical slot having radius $r_{1}$ mm is introduced as shown in Fig. 2a. A ring with radius $r_{2}$ mm is subsequently introduced around this square, housing one slot of size *s* mm as shown in Fig. 2a. This unit cell pattern is astutely selected among various shapes to facilitate the desired multi-functional outcome, particularly in reflection mode. To analyze the unit cell’s performance, a single instance is simulated with periodic boundary conditions along *y*-directions. EM software CST Microwave $\text {Studio}^{\circledR }$ is employed to assess co-polarization and cross-polarization conversions across the frequency range of 10–60 GHz. Floquet ports are applied to the periodic structure to scrutinize its reflection characteristics.

### **Theoretical analysis**

**Fig. 3:**
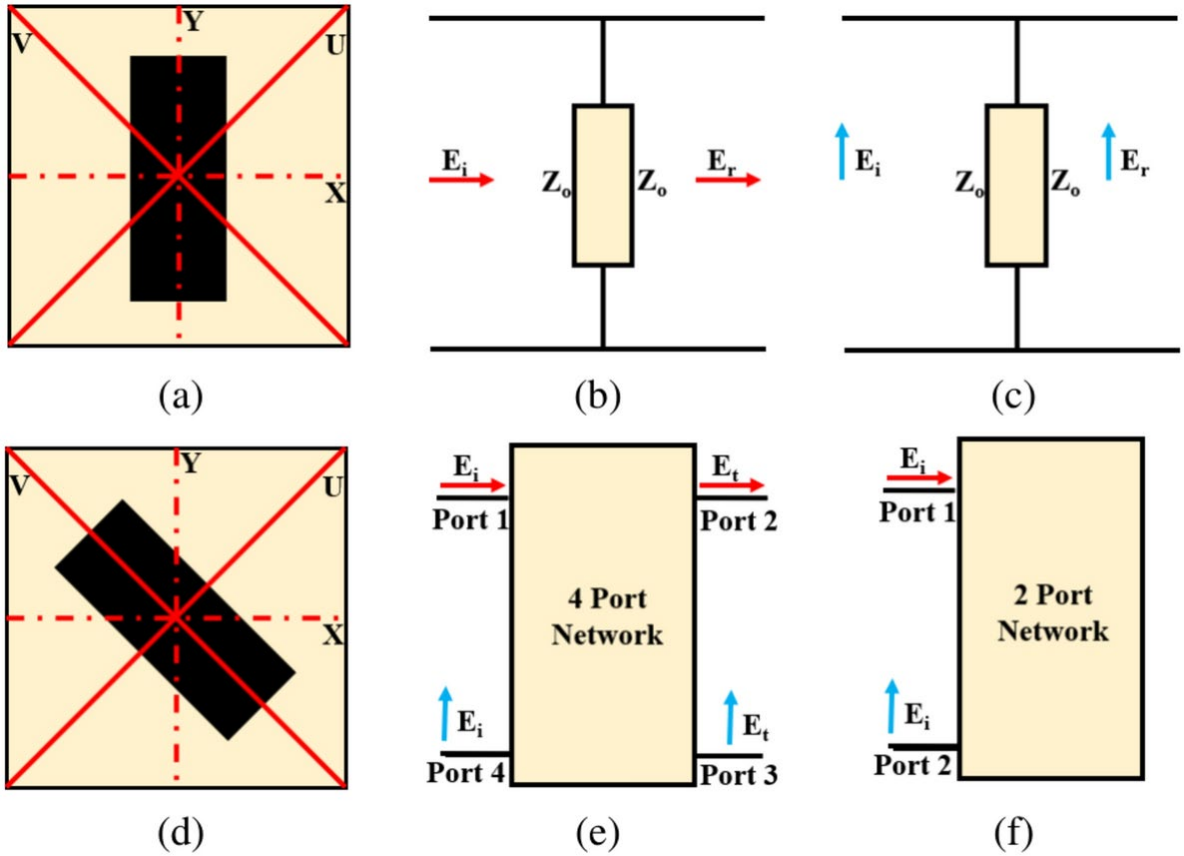
The unit cell model (**a**) symmetry along X- and Y-axis (**b**) four port network model of symmetric unit cell for horizontal polarization (**c**) four port network model of symmetric unit cell for vertical polarization (**d**) asymmetry along X- and Y-axis (**e**) four port asymmetric unit cell (**f**) two port network model of asymmetric unit cell.

To gain insights into the polarization conversion mechanism, it is essential to look into the physical underpinnings of polarization conversion. Let’s begin by considering a square-shaped unit cell featuring a rectangular metallic strip. It is important to note that, for the sake of simplicity, we assume negligible metallization thickness. This structure is positioned atop a dielectric substrate and exhibits symmetry along both the X and Y axes. The conceptual diagram is presented in Fig. 3a.

Upon examination, it becomes evident that this unit cell showcases symmetries in both the horizontal (H) and vertical (V) planes. When an incident EM wave with normal incidence has its electric field polarized along the horizontal axis or X-axis, it generates no vertical or Y-axis component in the reflecting or transmitting wave. A similar behavior is observed when the EM wave is polarized along the vertical axis or Y-axis. Consequently, both polarizations remain decoupled and can be independently described.

In light of this, it becomes apparent that the equivalent circuit of the unit cell depicted in Fig. 3a must be separately articulated for horizontal and vertical polarization, as illustrated in Fig. 3b, c. In summary, based on the preceding discussion, it is evident that the unit cell shown in Fig. 3a is ill-suited for effecting polarization conversion of EM waves.

Moving forward, our analysis turns to a unit cell characterized by asymmetry along both the X and Y axes, exemplified in Fig. 3d. In this configuration, the cell is capable of generating both horizontal and vertical components of the electric field. The unit cell depicted in Fig. 3d can be conceptualized as a four-port network, as visualized in Fig. 3e. Each port within this network corresponds to a specific polarization, encompassing both horizontal and vertical polarization.

However, in the context of a reflective-type polarization converter, there is minimal transmission of EM waves from the input port to the output port. Consequently, the four-port network model is streamlined into a two-port network, illustrated in Fig. 3f. This transformation is driven by the inherent nature of reflective converters, where the focus is primarily on reflection rather than transmission.

The S matrix of the copper-backed reflective polarization converter can be mathematically expressed as:

1 $$\begin{aligned} \text {S} = \begin{bmatrix} \text {S}_{11} & \text {S}_{12} \\ \text {S}_{21} & \text {S}_{22} \end{bmatrix} \end{aligned}$$

In the context of Eq. (1), $\text {S}_{11}$ represents the co-polarized reflection coefficient ($\text {R}_{yy}$), while $\text {S}_{21}$ signifies the cross-polarized reflection coefficient ($\text {R}_{xy}$). The manipulation of this coefficient’s magnitude allows us to achieve the desired polarization conversion using a metasurface. Building upon the explanation presented earlier, the process of accomplishing polarization conversion with a metasurface can also be envisioned as a linear time-invariant system (LTI).

In this framework, the input vector corresponds to the incident EM wave, which undergoes transformation into the output vector-the polarization-converted reflected EM wave. Consequently, the incident electric field, designated as $[\text {E}_{ix} \hspace{3pt} \text {E}_{iy}]^\text {T}$, and the reflected electric field, represented as $[\text {E}_{rx} \hspace{3pt} \text {E}_{ry}]^\text {T}$, can be mathematically related as

2 $$\begin{aligned} \begin{bmatrix} \text {E}_{rx} \\ \text {E}_{ry} \end{bmatrix} = \begin{bmatrix} r_{xx} & r_{xy} \\ r_{yx} & r_{yy} \end{bmatrix} \begin{bmatrix} \text {E}_{ix} \\ \text {E}_{iy} \end{bmatrix} \end{aligned}$$

In the context of Eq. (2), $\text {R}_{ij}$ represents the reflection coefficient, where ’*j*’ signifies the incident linear polarization and ’*i*’ represents the reflected polarization. Therefore, for an ideal polarization converter, the co-polarization reflection coefficient and cross-polarization reflection coefficient are defined below in Eq. (3) as

3a $$\begin{aligned}&\text {R}_{xx}=\text {R}_{yy}\approx 0, \end{aligned}$$

3b $$\begin{aligned}&\text {R}_{yx}=\text {R}_{xy}\approx 1 \end{aligned}$$

Thus the reflection matrix for a polarization converter is defined below in Eq. (4) as

4 $$\begin{aligned} \text {S} = \begin{bmatrix} 0 & 1 \\ 1 & 0 \end{bmatrix} \end{aligned}$$

In the next step, we proceed to determine the eigenvalues and eigenvectors for the polarization-converting metasurface. The obtained eigenvectors are denoted as follows: $$u= \begin{bmatrix} 1&1 \end{bmatrix}^\text {T}$$ and $$v= \begin{bmatrix} -1&1 \end{bmatrix}^\text {T}$$, corresponding to eigenvalues $e^{i0}=1$ and $e^{i\pi }=-1$ respectively. In a physical context, this signifies that when the electric field aligns with the direction of either the $u-$ or $v-$eigenvectors, both are tilted by ${\mp }45^\circ$ to the *y*, axis-the reflected EM wave experiences no polarization rotation. As there is no polarization rotation along the *u*- and *v*-axes, we can conclude below in Eq. (5) as

5a $$\begin{aligned}&\text {R}_{uu}=\text {R}_{vv}\approx 1 \end{aligned}$$

5b $$\begin{aligned}&\text {R}_{uv}=\text {R}_{vu}\approx 0 \end{aligned}$$

Hence, when considering a normally incident *y*-polarized EM wave with an electric field denoted as $\text {E}_y = {\hat{y}}\text {E}_{i}e^{ikz}$, it can be expressed as the sum of two mutually orthogonal components in the directions of ${\hat{u}}$ and ${\hat{y}}$, represented as:

6 $$\begin{aligned} \text {E}_{i} = \left( {\hat{u}}\text {E}_{iu} + {\hat{v}}\text {E}_{iv}\right) e^{ikz}={\hat{y}}\text {E}_i \end{aligned}$$

In Eq. (6), both $\text {E}_{iu}$ and $\text {E}_{iv}$ are equal to $0.707E_i$. Since the components of the electric field along the *u*- and *v*-directions correspond to the eigenvectors, they are reflected with the same magnitude. Consequently, $\text {E}_{ru}$ and $\text {E}_{rv}$ are both equal to $0.707E_r$, with phase differences of $0^\circ$ and $180^\circ$ respectively. Therefore, the reflected field can be expressed as:

7 $$\begin{aligned} \text {E}_{r} = \left( {\hat{u}}\text {E}_{ru} - {\hat{v}}\text {E}_{rv}\right) e^{ikz}={\hat{x}}\text {E}_r \end{aligned}$$

From Eq. (6) and (7), it can be deduced that the incident *y*-polarized EM wave is reflected along the x-axis, resulting in a polarization rotation of $90^\circ$. This phenomenon is visually depicted in Fig. 2c, where the reflected electric field $\text {E}_r$, obtained from the vector sum of $\text {E}_{ru}$ and $\text {E}_{rv}$, aligns with the x-axis. As previously discussed, for cross-polarization conversion, the phase difference between ${\hat{u}}$ and ${\hat{v}}$ should be an odd multiple of $180^\circ$, i.e., $\Delta _{uv}=arg(\text {R}_{uu})-arg(\text {R}_{vv})=n\pi$, where ’n’ is an odd integer.

By combining Eq. (6) with Eq. (7), it can be asserted that the electric field of the reflected wave can be expressed in Eq. (8) as:

8 $$\begin{aligned} \text {E}_{r}=\left( {\hat{u}}\text {R}_{uu}\text {E}_{iu}e^{i\phi _{uu}}-{\hat{v}}\text {R}_{vv}\text {E}_{iv}e^{i\phi _{vv}}\right) e^{ikz} \end{aligned}$$

Since the reflected polarization field is perpendicular to the incident EM wave, the dot product of $\text {E}_{i}$ and $\text {E}_{r}$ equals zero at $z=0$, and is given in Eq. (9) as

9 $$\begin{aligned} \text {E}_{iu}e^{i\phi _{uu}}(1+e^{i\Delta \phi })=0 \end{aligned}$$

This condition can only be met if $\Delta \phi =n\pi$, where *n* takes on values such as $\pm 1,\pm 3,\cdots$.

## **Simulation and analysis**

To thoroughly evaluate the effectiveness of the proposed metasurface in polarization conversion, we conducted numerical simulations using the EM software CST Microwave $\text {Studio}^\circledR$. These simulations incorporated Floquet ports to accurately excite the structure and employed master-slave boundary conditions to simulate an infinite array scenario. A fine mesh grid was applied under periodic boundary conditions, yielding consistent results that confirm simulation convergence. The unit cell simulation utilized a general-purpose frequency domain solver, with open boundary conditions implemented via Floquet port configurations. Additionally, Floquet port Zmax was set with two Floquet modes enabled to enhance accuracy and performance across the frequency range.

When a linearly polarized EM wave with an electric field component in the y-direction is incident, it can reflect either in the same direction (co-polarization) as defined in Eq. (3a) or in the opposite direction (cross-polarization) as defined in Eq. (3b). This principle also applies to *x*-polarized incident EM waves. Therefore, $\text {R}_{xx} = \text {E}_{rx}/\text {E}_{ix}$ and $\text {R}_{yy} = \text {E}_{ry}/\text {E}_{iy}$ represent the co-polarized reflection parameters, while $\text {R}_{yx} = \text {E}_{ry}/\text {E}_{ix}$ and $\text {R}_{xy} = \text {E}_{rx}/\text {E}_{iy}$ represent the cross-reflection parameters. To simplify our study, we will continue with a linearly polarized EM wave with its electric field in the y-direction.

### **Linear polarization**

**Fig. 4:**
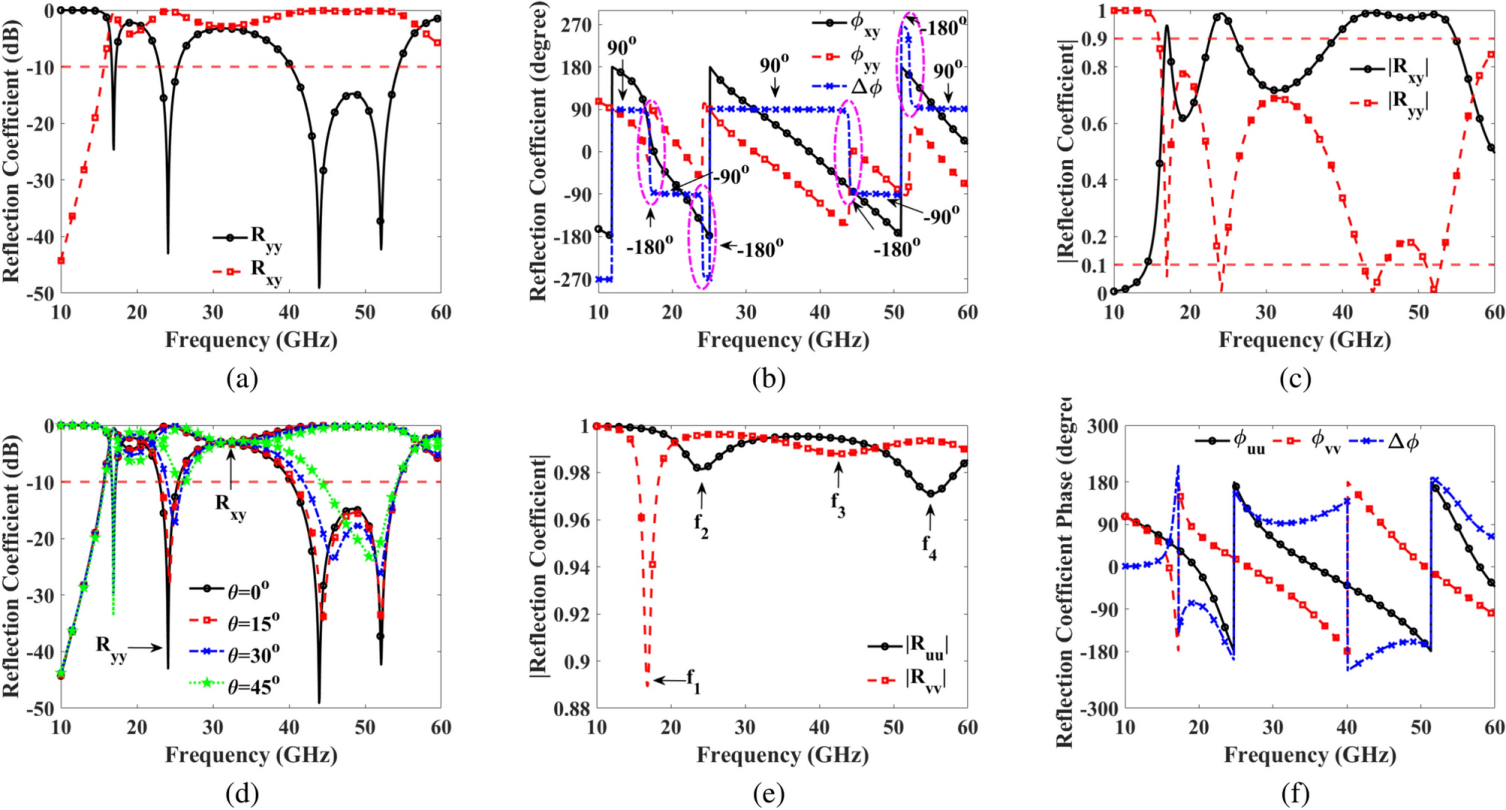
Simulation results (**a**) co-reflection ($\text {R}_{yy}$) and cross-reflection ($\text {R}_{xy}$) coefficients in dB (**b**) the phase and phase difference of co- and cross-reflection coefficients in degree (**c**) the magnitude of co- and cross-reflection coefficients (**d**) co- and cross-reflection coefficients (dB) under normal, and oblique incident electromagnetic wave (**e**) the magnitude of co- and cross-reflection coefficients in *u* and *v* directions (**f**) the phase and phase difference (degree) of $\text {R}_{uu}$ and $\text {R}_{vv}$.

Figure 4a depicts the co-reflection and cross-reflection coefficients in decibels (dB). The co-reflection coefficients remain below -10 dB, while the cross-reflection coefficients are above -1 dB across the frequency ranges of 16.7-17.2 GHz, 23.0-25.4 GHz, and 40.3-54.35 GHz, with resonances observed at 16.95 GHz, 24.1 GHz, 43.95 GHz, and 52.1 GHz. The co-reflection and cross-reflection coefficients are critical indicators of polarization conversion efficiency. In the specified frequency ranges (16.7-17.2 GHz, 23.0-25.4 GHz, and 40.3-54.35 GHz), low co-reflection coefficients below -10 dB demonstrate effective suppression of original polarization, while high cross-reflection coefficients above -1 dB signify strong polarization conversion, indicating superior performance at resonant frequencies. Additionally, the relative phase difference between $\text {R}_{yy}$ and $\text {R}_{xy}$ at these resonating frequencies is also monitored. The relative phase difference is calculated as:

10 $$\begin{aligned} \Delta \phi =\text {arg}(\text {R}_{xy})-\text {arg}(R_{yy}), \end{aligned}$$

The significance of polarization can be understood from Eq. (10), where if $\Delta \phi =0, \pm \pi$, it indicates LP^32^. Figure 4b clearly shows that at frequencies 16.95 GHz, 24.1 GHz, 43.95 GHz, and 52.1 GHz, the phase differences are approximately -$180^\circ$, -$180^\circ$, -$180^\circ$, and -$180^\circ$, respectively. The near 180-degree phase difference between co- and cross-reflection coefficients signifies efficient LP conversion in the specified frequency ranges. This phase shift ensures that the incident wave’s polarization is accurately rotated, making the metasurface highly effective for LP conversion, crucial for applications requiring precise polarization control. A summary of the data for LP is presented in Table 1.

**Table 1:** Linear polarization.

Band	Freq (GHz)	BW (GHz)	Resonance (GHz)	$\mathbf {\Delta \phi }$	PCR
Ku	16.7-17.2	0.50	16.95	-$180^\circ$	$\ge 90\%$
K	23.0-25.4	2.40	24.10	-$180^\circ$	$\ge 90\%$
U	40.3-54.35	14.05	43.95, 52.1	-$180^\circ$	$\ge 90\%$

Figure 4c illustrates the amplitudes of the co-reflection coefficient, $\text {R}_{yy}$, and the cross-reflection coefficients, $\text {R}_{xy}$, under normal incidence. The graph clearly shows that $\text {R}_{yy}$ approaches 0.1, while $\text {R}_{xy}$ reaches 0.9 within the frequency ranges of 16.7-17.2 GHz, 23.0-25.4 GHz, and 40.3-54.35 GHz. Additionally, at resonance frequencies of 16.95 GHz, 24.1 GHz, 43.95 GHz, and 52.1 GHz, the co-reflection coefficient nearly vanishes, while the cross-reflection coefficient approaches 1. The behavior of the co- and cross-reflection coefficients, with the co-reflection coefficient approaching 0 and the cross-reflection coefficient nearing 1 at resonance frequencies, indicates highly effective LP conversion. This suggests that the metasurface efficiently redirects the incident wave’s polarization, making it ideal for polarization control in LP applications. Furthermore, Fig. 4d displays the co-reflection and cross-reflection coefficients in dB under oblique incidence. It is evident that the co-reflection and cross-reflection coefficients remain within the -10 dB and -1 dB regions, respectively, up to $45^\circ$.

**Fig. 5:**
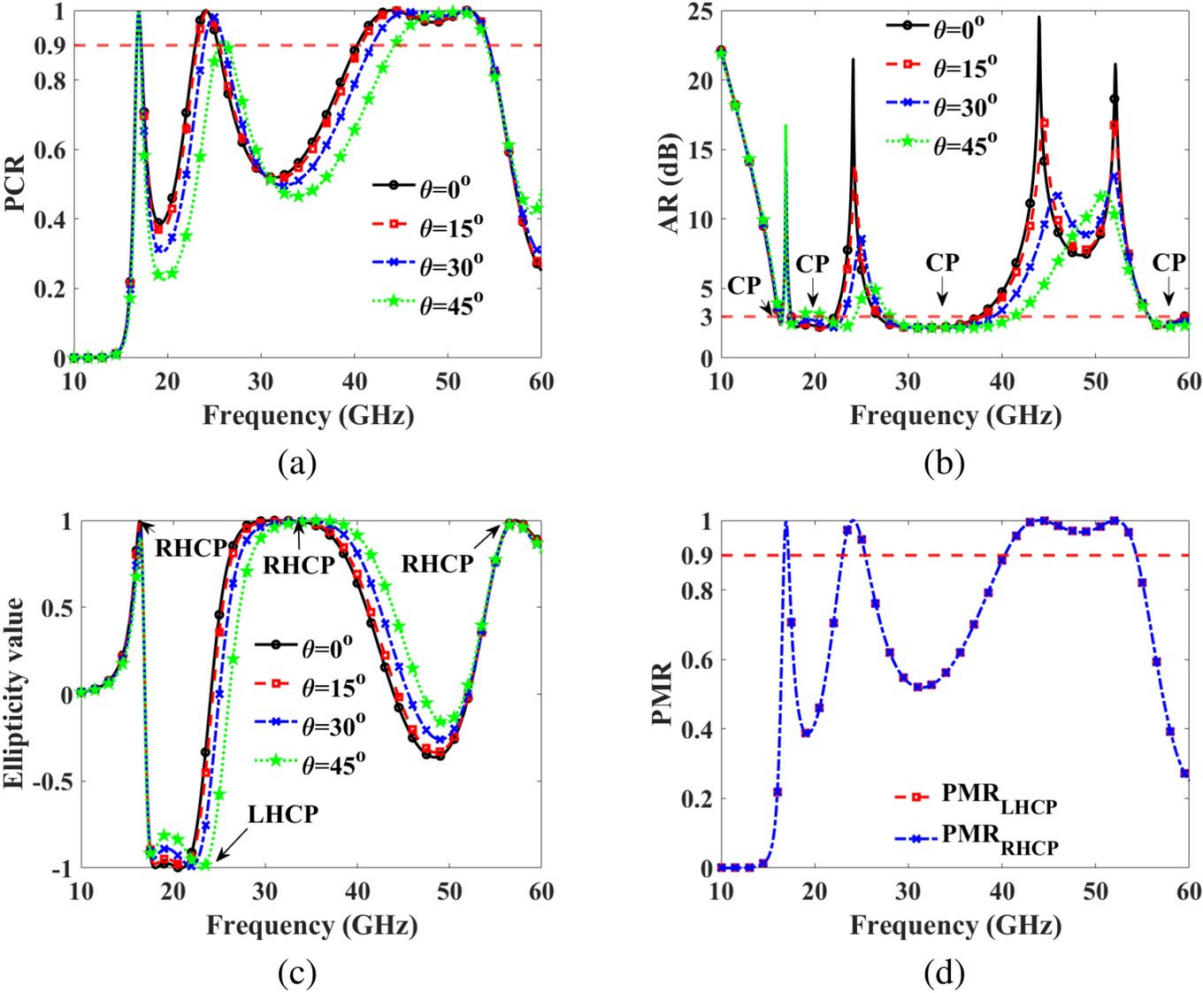
Simulation results (**a**) PCR under oblique incidence (**b**) axial ratio under oblique incidence (**c**) ellipticity (**d**) PMR for RHCP, and LHCP incidence EM wave.

To verify the effectiveness of the proposed metasurface polarization converter in cross-polarization scenarios, we analyze its response to incident EM waves polarized along the u- and v-directions. In Fig. 2c, the u- and v-axes are oriented at angles of ${\mp }45^\circ$ relative to the y-axis. The magnitudes of the reflection coefficients in the u- and v-directions, along with the phase difference analysis of these coefficients, are presented in Fig. 4e, f. Figure 4e reveals that the magnitudes of the reflection coefficients ($\text {R}_{uu}$, $\text {R}_{vv}$) in the u- and v-directions are approximately 1 across the entire frequency bandwidth from 10 to 60 GHz. These directional reflection coefficients exhibit resonance at four dip points. At these dip points, eigenmodes are excited at frequency $f_2=24.1$ GHz and $f_4=55.15$ GHz with $\text {E}_u$, while eigenmodes are excited at frequencies $f_1=16.85$ GHz and $f_3=42.75$ GHz with $\text {E}_v$, similar to the observations in Ref.^33^. Additionally, in Fig. 4f, the phase differences of the reflection coefficients in the u- and v-directions are approximately -$180^\circ$, and ${\mp }90^\circ$ where LP, CP occurs, respectively, within the same frequency region. Hence, it is confirmed that the metasurface is capable of LP and CP conversion within the relevant bandwidth.

To assess the performance of the LP converter, the PCR^22^ for both x- and y-polarized incident EM waves can be calculated using below Eq. (11):

11 $$\begin{aligned} \text {PCR}=\frac{\text {R}_{yx}^2}{\text {R}_{yx}^2+\text {R}_{xx}^2} = \frac{\text {R}_{xy}^2}{\text {R}_{xy}^2+\text {R}_{yy}^2}. \end{aligned}$$

The PCR for the y-polarized incident EM wave of the proposed metasurface is determined using Eq. (11), as depicted in Fig. 5a, under both normal and oblique incidences. As illustrated, the PCR value of the metasurface exceeds 90% under normal incidence within the frequency range of 16.7-17.2 GHz, 23.0-25.4 GHz, and 40.3-54.35 GHz. Moreover, under oblique angle performance, over 90% PCR is achieved up to $45^\circ$.

### **Circular polarization**

To analyze the CP conversion capability of the metasurface, it is essential to remember that for achieving CP ideally, the amplitudes of the two mutually orthogonal fields should be equal, and their phase difference must be an odd multiple of $90^\circ$, i.e.$\Delta \phi = n\pi /2$, where n is an odd integer. Figure 4b displays the phase difference between the co- and cross-polarized reflection coefficients. The AR considers both the amplitudes and phase differences of the reflected fields, defined as:

12 $$\begin{aligned} \text {AR}=\frac{1}{2}\left( \frac{|\text {R}_{yy}|^2+|\text {R}_{xy}|^2+\sqrt{|\text {R}_{yy}|^4+|\text {R}_{xy}|^4+2|\text {R}_{yy}|^2|\text {R}_{xy}|^2\text {cos}(2\Delta \phi )}}{|\text {R}_{yy}|^2+|\text {R}_{xy}|^2+\sqrt{|\text {R}_{yy}|^4+|\text {R}_{xy}|^4+2|\text {R}_{yy}|^2|\text {R}_{xy}|^2\text {cos}(2\Delta \phi )}}\right) ^\frac{1}{2}, \end{aligned}$$

The AR indicates CP when it is less than or equal to 3 dB, i.e., $AR \le 3$ dB^32^. Figure 5b illustrates the AR obtained from Eq. (12). It is evident from the figure that the CP criterion ($AR \le 3$ dB) is met within four frequency ranges: 16.1-16.55 GHz, 17.5-22.15 GHz, 26.65-37.75 GHz, and 55.6-59.8 GHz. The co- and cross-reflection coefficients above 5 dB in the frequency ranges of 16.1-16.55 GHz, 17.5-22.15 GHz, 26.65-37.75 GHz, and 55.6-59.8 GHz indicate strong reflective performance, ensuring efficient signal conversion. With the AR below 3 dB in these ranges, the design achieves high-fidelity CP conversion. This combination enhances the effectiveness of the metasurface for applications requiring stable polarization control across diverse satellite communication bands.

To analyze the handedness of the reflected circular polarization (CP), we utilize Stokes’ parameters^32^, which are defined in Eq. (13) as:

13a $$\begin{aligned}&\text {S}_0=|\text {R}_{yy}|^2+|\text {R}_{xy}|^2, \end{aligned}$$

13b $$\begin{aligned}&\text {S}_1=|\text {R}_{yy}|^2-|\text {R}_{xy}|^2, \end{aligned}$$

13c $$\begin{aligned}&\text {S}_2=2|\text {R}_{yy}||\text {R}_{xy}|\text {cos}(\phi ), \end{aligned}$$

13d $$\begin{aligned}&\text {S}_3=2|\text {R}_{yy}||\text {R}_{xy}|\text {sin}(\phi ), \end{aligned}$$

The normalized ellipticity, denoted as $e = \text {S}_3/\text {S}_0$, signifies the polarization state. It is evident from Stokes’ parameters that the normalized ellipticity is +1 for RHCP and -1 for LHCP. The outcomes depicted in Fig. 5c reveal that the ellipticity is 1, indicating RHCP for three frequency bands: 16.1-16.55 GHz, 26.65-37.75 GHz, and 55.6-59.8 GHz. Conversely, the ellipticity is -1, indicating LHCP in one frequency band: 17.5-22.15 GHz.

RHCP is widely used in Ku, Ka, and U bands for satellite applications due to its ability to minimize signal degradation caused by atmospheric effects such as rain and multi-path fading. In the Ku-band (16.1-16.55 GHz), RHCP is commonly utilized for satellite TV broadcasting and data communications. In the Ka-band (26.65-37.75 GHz), RHCP supports high-speed satellite internet and broadband services, offering higher frequency spectrum for better data throughput. For U-band (55.60-59.8 GHz), RHCP enhances communication in advanced satellite systems, ensuring efficient transmission and reception in higher frequency applications with minimal interference. LHCP is used in Ku, K (17.5-22.15 GHz) bands for satellite applications to improve signal clarity and reduce interference. It helps in achieving robust communication links, particularly in satellite TV broadcasting, data transmissions, and military communication systems, where polarization diversity is essential.

In addition to examining the orthogonal polarization conversion of linearly polarized electromagnetic (EM) waves, we also need to explore the response of the proposed reflective polarization converter when subjected to incident circularly polarized EM waves^27^. In the preceding discussion of this section, we have already determined the magnitude and phase of the reflection coefficient of the metasurface based on a rectangular basis. These responses can be readily converted into circular basis using.

14a $$\begin{aligned} \text {R}_{cp}&= \begin{bmatrix} \text {R}_{RR} & \text {R}_{RL} \\ \text {R}_{LR} & \text {R}_{LL} \end{bmatrix}, \end{aligned}$$

14b $$\begin{aligned}&= \frac{1}{2}\begin{bmatrix} \text {R}_{xx}-\text {R}_{yy}-i(\text {R}_{xy}+\text {R}_{yx}) & \text {R}_{xx}+\text {R}_{yy}+i(\text {R}_{xy}-\text {R}_{yx}) \\ \text {R}_{xx}+\text {R}_{yy}-i(\text {R}_{xy}+\text {R}_{yx}) & \text {R}_{xx}-\text {R}_{yy}+i(\text {R}_{xy}+\text {R}_{yx}) \end{bmatrix} . \end{aligned}$$

In this equation, the subscript ‘*R*’ indicates the RHCP wave, whereas the subscript ‘*L*’ indicates the LHCP wave. It should be noted that when an RHCP-polarized wave encounters a metallic reflector or mirror, it is reflected as an LHCP wave, and vice versa. From Eq. (14), it is observed that when $\text {R}_{xx}=\text {R}_{yy} = 0$ and $\text {R}_{xy}=\text {R}_{yx} = 1$, then $\text {R}_{RR}=\text {R}_{LL} = 1$. Hence, based on the derived equation, it can be inferred that when a circularly polarized wave impinges upon the metasurface, the resulting reflected wave preserves the essential characteristics of polarization at frequencies corresponding to 16.95 GHz, 24.1 GHz, 43.95 GHz, and 52.1 GHz. This highlights the polarization maintaining capability, which is further quantified by defining the polarization maintaining ratio (PMR) in Eq. (15) as

15 $$\begin{aligned} \text {PMR}=\frac{\text {R}_{LR}^2}{\text {R}_{LR}^2+\text {R}_{RR}^2} = \frac{\text {R}_{RL}^2}{\text {R}_{RL}^2+\text {R}_{LL}^2}. \end{aligned}$$

Based on the aforementioned analysis, it can be affirmed that the Polarization Maintaining Ratio (PMR) for circular polarization mirrors the Polarization Conversion Ratio (PCR) observed in the orthogonal polarization conversion band. Consequently, within these frequency bands, the reflection coefficients $\text {R}_{RR}=\text {R}_{LL} = \text {R}_{xy}=\text {R}_{yx} = 1$, while the remaining coefficients $\text {R}_{LR}=\text {R}_{RL} = \text {R}_{xx}=\text {R}_{yy} = 0$. This indicates that the proposed structure can effectively function as a meta-reflector, preserving the handedness of CP upon reflection at frequencies corresponding to 16.95 GHz, 24.1 GHz, 43.95 GHz, and 52.1 GHz.

Eq. (15) gives the LHCP and RHCP PMR under the RHCP and LHCP incidence EM waves. Figure 5d illustrates the LHCP and RHCP, respectively, PMR of the proposed metasurface under RHCP and LHCP incidence EM waves. It is observed that RHCP and LHCP are also achieved when the metasurface is subjected to LHCP and RHCP incidence EM waves, respectively, for the three frequency bands. A summary of the results for CP is provided in Table 2.

**Table 2:** Circular polarization.

Band	Freq (GHz)	BW (GHz)	Ellipticity value	$\Delta \phi$	Type of CP
Ku	16.1-16.55	0.45	$+1$	$90^\circ$	RHCP
Ku, K	17.5-22.15	4.65	$-1$	$-90^\circ$	LHCP
Ka	26.65-37.75	11.10	$+1$	$+90^\circ$	RHCP
U	55.60-59.8	4.20	$+1$	$+90^\circ$	RHCP

## **Design evolution and parametric study**

### **Design evolution**

Designing metasurfaces for polarization conversion across the Ku, K, Ka, and U bands requires careful tuning of the unit cell geometry to deliver the desired EM response over multiple frequency ranges. This involves selecting materials with suitable electric and magnetic properties, and creating resonant structures, such as ring with slot and a square with slot, that are optimized for size, orientation, and periodicity. Achieving polarization conversion hinges on inducing differential phase shifts between the orthogonal components of the incoming EM wave, ensuring a high PCR over wide bandwidths. Furthermore, the metasurface must be resilient to oblique incident angles, making it well-suited for practical applications like Satcom and remote sensing.

In this study, we designed a metasurface unit cell with dimensions optimized for polarization conversion. The unit cell has a length of 2 mm and a thickness of 0.76 mm, allowing it to operate effectively within a broad frequency range from 10 to 60 GHz, with a central frequency at $f_\circ =35$ GHz. The length of the unit cell is specifically chosen to be $0.23 \times \lambda _\circ$ at this central frequency, which influences its ability to resonate at multiple frequencies. Resonance is observed at $f_1=16.95$ GHz, $f_2=24.1$ GHz, $f_3=43.95$ GHz, and $f_4=52.1$ GHz, where corresponding length of the unit cell is $0.11 \times \lambda _1$, $0.16 \times \lambda _2$, $0.29 \times \lambda _3$, and $0.34 \times \lambda _4$, respectively. This highlights the unit cell’s capability to support multiple resonances due to its compact size and optimized design. The relationship between the unit cell’s physical dimensions and the resonant frequencies is critical in achieving effective polarization conversion across the working band. By leveraging this dimension-frequency correlation, the metasurface exhibits strong resonance, enhancing its efficiency in polarization conversion at the desired frequency bands.

The design process involves three main steps: Firstly, creating a ring with a slot ensures the rotation of the incident LP incident EM wave to its orthogonal LP reflected EM wave, facilitating LP and CP conversion in the lower and higher frequency bands. Secondly, designing a square with a slot and rotating it at $45^\circ$ disrupts the polarization states to achieve multi-band LP in the upper frequency band. Lastly, parameter optimization is conducted to enable the reflection of LP EM waves in the three bands and CP EM waves in the four different sub-bands.

**Fig. 6:**
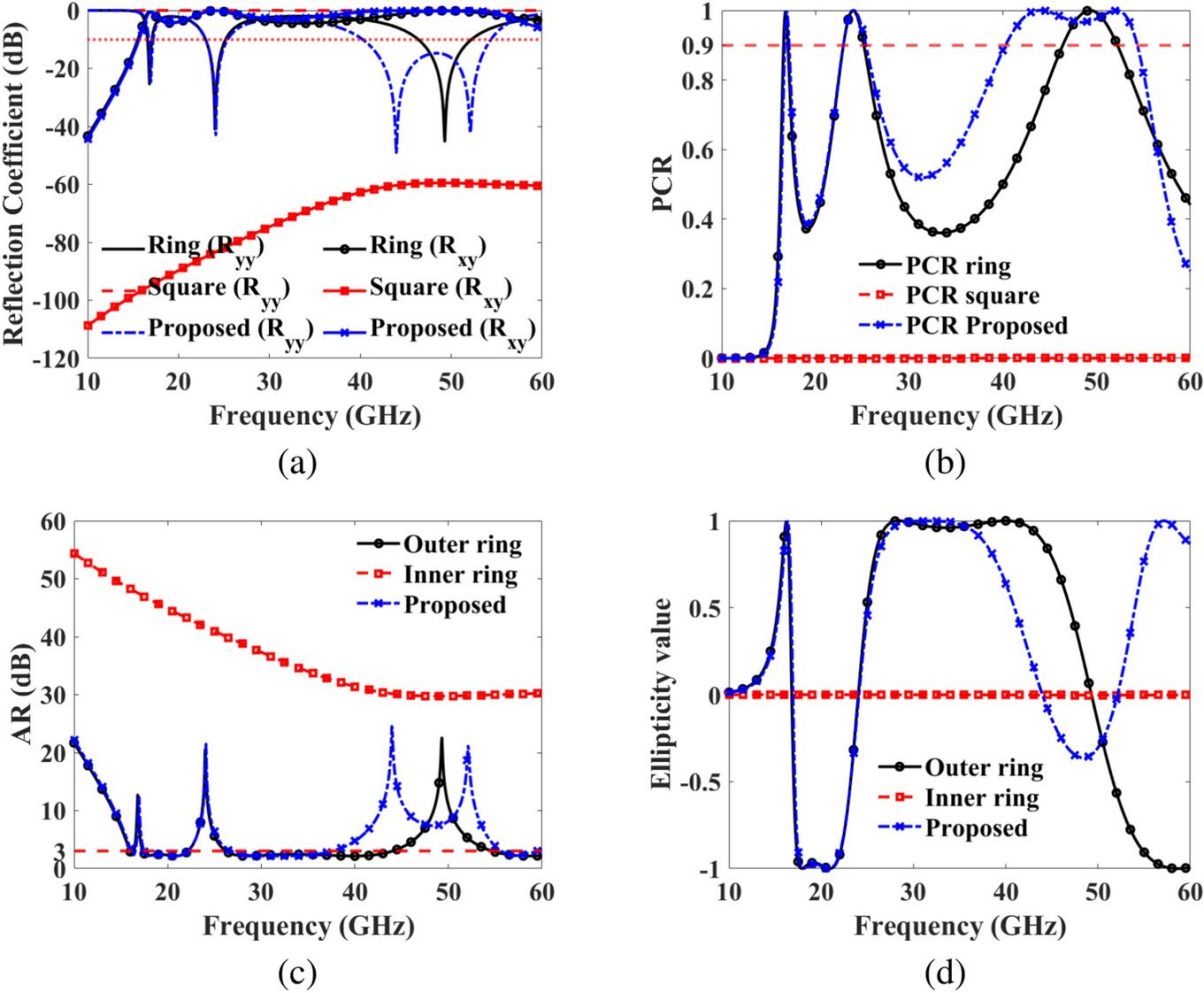
Design evolution (**a**) Reflection coefficient (**b**) PCR (**c**) Axial ratio (**d**) Ellipticity.

The reflection coefficients, covering both co-polarization and cross-polarization, alongside the PCR of the ring, square, and the proposed structure, are depicted in Fig. 6a, b. The ring exhibits co-polarization ($\text {R}_{yy}$) below -10 dB within the frequency bands of 16.6-17.05 GHz, 23-25.15 GHz, and 46.4-52.2 GHz, achieving a PCR $\ge 0.90$ in these ranges. This performance peaks at 99.97% at the resonance frequencies of 16.8 GHz and 24 GHz, and 49.3 GHz. Conversely, the square demonstrates co-polarization ($\text {R}_{yy}$) near 0 dB from 10-60 GHz, while simultaneously displaying cross-polarization ($\text {R}_{xy}$) below -60 dB within the 10-60 GHz range. Thus, the co- and cross-polarized reflection coefficients of the square are engineered to disturb polarization states and achieve multi-band LP in the higher frequency range. When the ring and square are integrated into a single metasurface, the proposed structure exhibits co-polarization ($\text {R}_{yy}$) below -10 dB in three frequency ranges from 16.7-17.2 GHz, 23-25.4 GHz, and 40.3-54.35 GHz. It achieves a PCR $\ge 0.90$ in these frequency ranges, peaking at 99.97% at the resonance frequencies of 16.95 GHz, 24.1 GHz, 43.95 GHz, and 52.1 GHz.

In Fig. 6c, d, we present the AR and ellipticity values across frequency bands for the Ring, Square, and the proposed structure. Figure 6c illustrates that the AR values for the ring structure remain below 3 dB between 15.95-16.45 GHz, 17.35-22.2 GHz, and 26.2-44.15 GHz, indicating CP conversion in the lower and higher frequency bands. However, for the square structure, the AR values stay above 29.75 dB from 10 to 60 GHz. In contrast, the proposed structure maintains AR values below 3 dB across wider frequency bands: 16.1-16.55 GHz, 17.5-22.15 GHz, 26.65-37.75 GHz, and 55.6-59.8 GHz, demonstrating CP conversion capability across the lower, middle, and higher bands. Figure 6d illustrates LHCP in 17.5-22.15 GHz while RHCP in 16.1-16.55 GHz, 26.65-37.75 GHz, and 55.6-59.8 GHz frequency ranges.

### **Parametric study**

A detailed parametric study was carried out to investigate the influence of various structural parameters on the PCR of the metasurface. Parameters such as ring dimensions, slot width, and rotation angles were adjusted to optimize the polarization conversion performance across different frequency bands. The ring size and slot dimensions were found to significantly affect the resonance frequencies, as larger rings shifted the resonance to lower frequencies while smaller slots narrowed the bandwidth. The rotation angle of the square structure influenced the polarization state by disrupting the symmetry, improving wide-band LP conversion in the upper frequency ranges. In contrast, fine-tuning the spacing between the meta-atoms helped to control the electric and magnetic dipole interactions, enhancing cross-polarization effects. Through this parametric study, it was observed that the PCR improved notably in specific frequency bands, confirming that precise adjustments to geometric parameters play a pivotal role in achieving optimal polarization conversion across the desired spectrum.

**Fig. 7:**
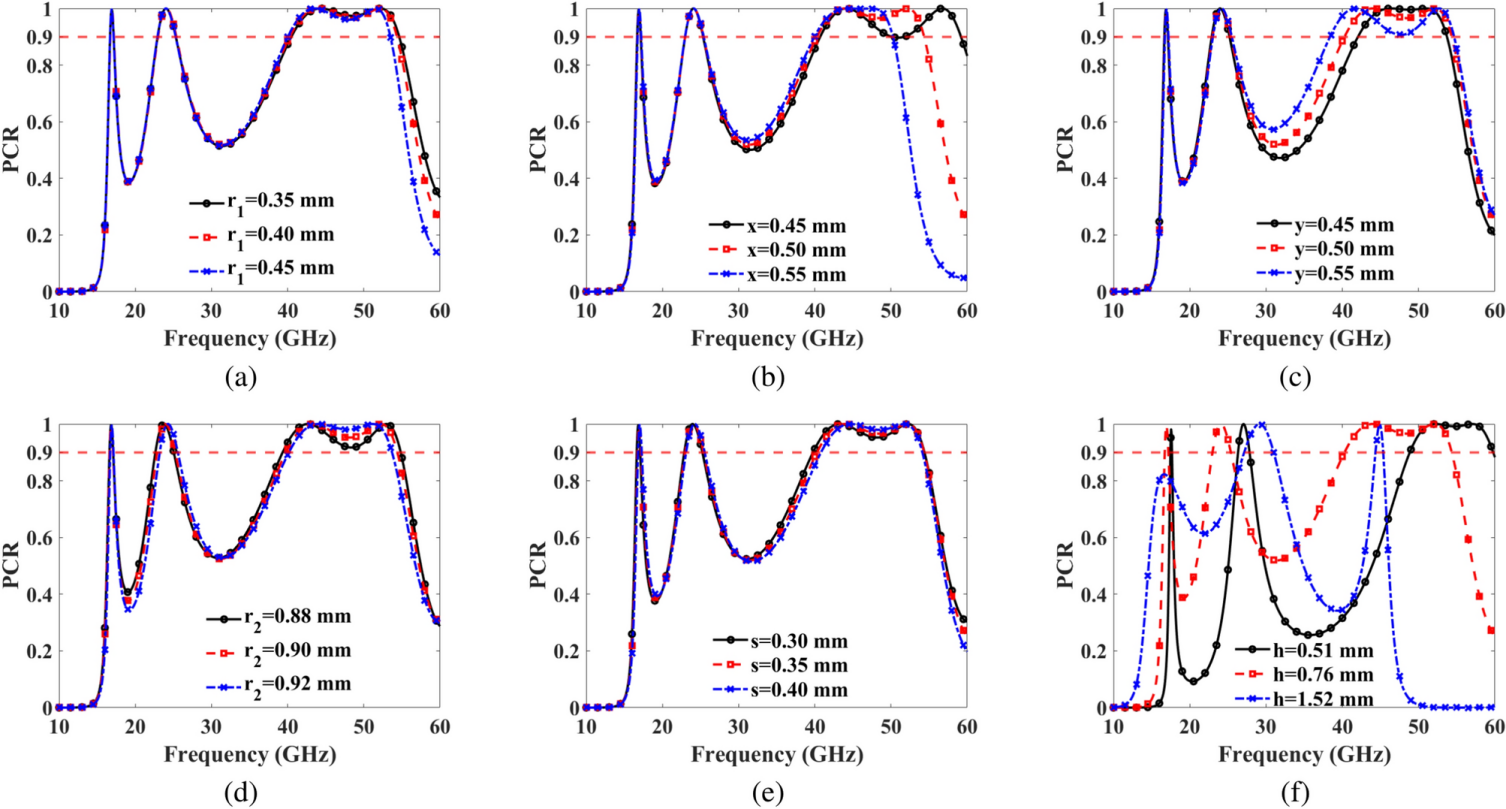
PCR of proposed metasurface (**a**) for $r_1=0.35-0.45$ mm (**b**) for $x=0.45-0.55$ mm (**c**) for $y=0.45-0.55$ mm (**d**) for $r_2=0.88-0.92$ mm (**e**) for $s=0.30-0.40$ mm (**f**) for $h=0.51-1.52$ mm.

The PCR variation, as per Eq. (11), is depicted in Fig. 7a–f, concerning detailed geometrical structures. To achieve effective LP with high PCR in the Ku, K, and U bands, and CPs (LHCP and RHCP) in the Ku, K, Ka and U bands, parameters were swept within the ranges: $r_1=0.35-0.45$ mm, $x=0.45-0.55$ mm, $y=0.45-0.55$ mm, $r_2=0.88-0.92$ mm, $s=0.30-0.40$ mm, and $h=0.51-1.52$ mm. From Fig. 7a–f, it is evident that the most effective PCR is achieved at $r_1=0.40$ mm, $x=0.50$ mm, $y=0.50$ mm, $r_2=0.90$ mm, $s=0.35$ mm, and $h=0.76$ mm.

The design of the concentric rings plays a pivotal role in achieving both LP and CP, including LHCP and RHCP. Key parameters like unit cell length *L* and substrate thickness *h* significantly influence the shift of LP and CP bands to lower or higher frequencies. The relationship between these dimensions and center frequencies is inversely proportional; as length or thickness increases, the operating frequency decreases. Fine-tuning the radii, $r_1$ and $r_2$, also offers additional control over band shifts, allowing precise adjustments to LP and CP bands. Their relationship with center frequencies follows an inverse pattern, similar to length and thickness. Moreover, modifying structural parameters such as lengths *x* and *y* and the slot width *s* is essential to maintain consistent PCR and effective LHCP and RHCP, particularly for oblique, *y*-polarized incident electromagnetic waves. These design optimizations enhance performance stability across a wide range of frequencies and incidence angles.

### **Surface current distributions**

To grasp the polarization conversion process, it is crucial to look into its underlying mechanism. When incident EM waves interact with meta-atoms, they induce electric and magnetic polarization within these meta-atoms. Consequently, electric and magnetic dipole moments are generated, which can interact with both electric and magnetic fields owing to the bi-anisotropic nature of the unit cell^21^. Eq. (16) elucidates the relationship between incident fields and the spatially averaged effective dipole moments.

16a $$\begin{aligned} p&=\begin{bmatrix} p_{x} \\ p_{y} \end{bmatrix} , \end{aligned}$$

16b $$\begin{aligned} m&=\begin{bmatrix} m_{x} \\ m_{y} \end{bmatrix}, \end{aligned}$$

16c $$\begin{aligned} \text {E}&=\begin{bmatrix} \text {E}_{x} \\ \text {E}_{y} \end{bmatrix}, \end{aligned}$$

16d $$\begin{aligned} \text {H}&=\begin{bmatrix} \text {H}_{x} \\ \text {H}_{y} \end{bmatrix}, \end{aligned}$$

16e $$\begin{aligned} \begin{bmatrix} p \\ m \end{bmatrix}&= \begin{bmatrix} \text {P}_{ee} & \text {P}_{em} \\ \text {P}_{me} & \text {P}_{mm} \end{bmatrix} \begin{bmatrix} \text {E} \\ \text {H} \end{bmatrix}, \end{aligned}$$

**Fig. 8:**
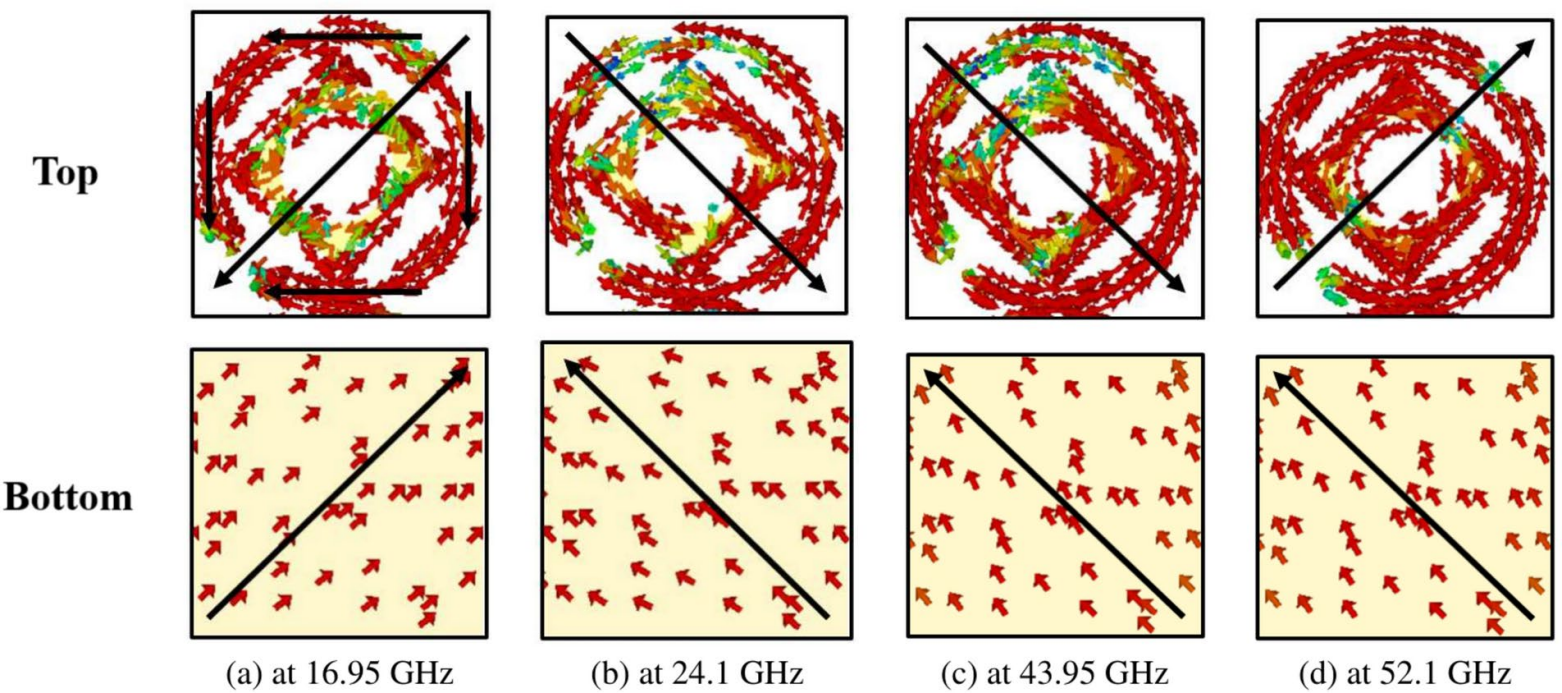
Surface current distribution on the top and bottom metal plate for linear polarization.

In the Eq. (16), the variables *p* and *m* symbolize the electric and magnetic dipole moments, respectively, whereas $\text {E}$ and $\text {H}$ represent the electric and magnetic fields, respectively. Additionally, $\text {P}_{em}$ signifies the electric-magnetic polarizability. The effective surface impedance is determined based on the electric and magnetic dipole moments of the meta-atoms.

17 $$\begin{aligned} Z_s(\omega )=\sqrt{\frac{\mu _{s}(\omega )}{\varepsilon _{s}(\omega )}}, \end{aligned}$$

In the Eq. (17), $\mu _{s}(\omega )$ and $\varepsilon _{s}(\omega )$ denote the frequency-dependent magnetic permeability and electric permittivity, respectively. Furthermore, the frequency-dependent reflection coefficient $\text {R}(\omega )$ can be determined using the surface impedance in the case of normal incidence, as illustrated in Eq. (18) below:

18 $$\begin{aligned} \text {R}(\omega )=\frac{\text {Z}_{s}(\omega )-\text {Z}_{\circ }}{\text {Z}_{s}(\omega )+\text {Z}_{\circ }}, \end{aligned}$$

In Eq. (18), where $\text {R}(\omega )$ is complex reflection coefficient with magnitude and phase, $\text {Z}_{\circ } = 120 \pi$ ohms represents the impedance of free space. The equation implies that $\text {R}(\omega _{r}) \approx 1$ when the surface impedance of the metasurface significantly surpasses the impedance of free space, $\text {Z}_{s}(\omega _{r}) \gg \text {Z}_{\circ }$, where $\text {R}(\omega )$ denotes the resonance frequency. In such cases, the structure functions as a High Impedance Surface (HIS) at specific frequencies, reflecting incident waves in phase with unity magnitude, unlike conventional reflectors that exhibit out-of-phase reversal. Expanding on this, when two orthogonal components of an incident field are reflected with $0^{\circ }$ and $180^{\circ }$ phases, the polarization plane of the wave rotates by $90^{\circ }$, leading to cross-conversion. This implies that the structure acts as an HIS for one component while operating as a common reflector for the other. To delve deeper into this concept, an examination of the surface current induced by time-varying dipole moments, generated by time-harmonic electric and magnetic incident waves, is necessary. This relationship is elucidated in Eq. (19):

19a $$\begin{aligned} \text {J}_s&=\begin{bmatrix} J_{x} \\ J_{y} \end{bmatrix} , \end{aligned}$$

19b $$\begin{aligned} \text {M}_s&=\begin{bmatrix} M_{x} \\ M_{y} \end{bmatrix} , \end{aligned}$$

19c $$\begin{aligned} \begin{bmatrix} J_{s} \\ M_{s} \end{bmatrix}&= i\omega \begin{bmatrix} \text {P}_{ee} & \text {P}_{em} \\ \text {P}_{me} & \text {P}_{mm} \end{bmatrix} \begin{bmatrix} E \\ H \end{bmatrix}. \end{aligned}$$

In Eq. (19), $\omega$ represents the angular frequency, while $\text {J}_s$ and $\text {M}_s$ denote the effective electric and magnetic surface current densities, respectively.

**Fig. 9:**
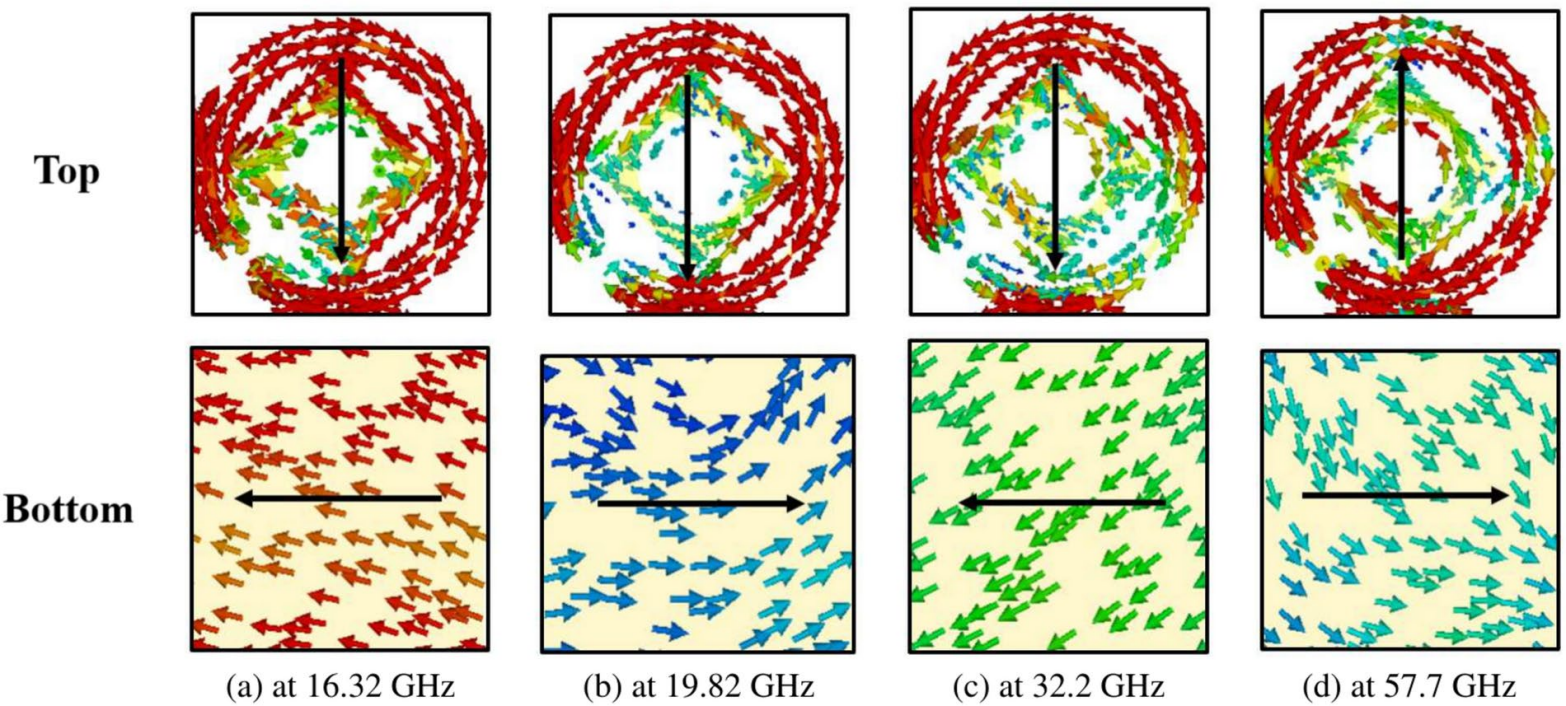
Surface current distribution on the top and bottom metal plate for circular polarization.

The induced Surface Current Distributions (SCD) at four resonance frequencies (16.95 GHz, 24.1 GHz, 43.95 GHz, and 52.1 GHz) are depicted in Fig. 8. At 16.95 GHz, as illustrated in Fig. 8a, distinct patterns are observed in the SCD on the top and bottom surfaces of the structure. In the ring component, surface vectors flow from the top-right to the bottom-left (indicated by solid black arrows), while on the bottom metallic plate, they flow from the left-bottom to the top-right (also indicated by solid black arrows). This indicates that the resonance at 16.95 GHz primarily manifests as magnetic, as evidenced by the anti-parallel surface current vectors on the top and bottom, enhancing the magnetic field within the dielectric substrate. This phenomenon is attributed to the increase in effective magnetic permeability, resulting in a significantly larger surface impedance compared to free space. Consequently, this satisfies the condition for a High Impedance Surface (HIS), $\text {Z}_{s}(\omega _{r}) \gg \text {Z}_{\circ }$. As a result, the phase reflection coefficient reaches unity magnitude, leading to a change in current flow towards the *x*-direction due to the phenomenon of impedance imbalance along the *y*-direction. Ultimately, this results in a $90^{\circ }$ polarization rotation, facilitating the reflection of the *x*-polarized wave from the surface^34^.

At 24.1 GHz, Fig. 8b SCD on the top and bottom surfaces of the structure. In both the ring and the square rotated at $45^{\circ }$, surface vectors flow from the top-left to the bottom-right (indicated by solid black arrows), while on the bottom metallic plate, they flow in the opposite direction, from bottom-right to top-left (also indicated by solid black arrows). This indicates that the resonance at 24.1 GHz primarily exhibits a magnetic nature, as evidenced by the anti-parallel surface current vectors on the top and bottom surfaces, enhancing the magnetic field within the dielectric substrate.

Furthermore, Fig. 8c depicts the SCD on the top and bottom surfaces of the structure at 43.95 GHz. Here, the surface vectors flow from bottom-right to top-left on both the ring and the square’s top surface (indicated by solid black arrows). Similarly, on the bottom metallic plate, the surface vectors also flow from bottom-right to top-left (as indicated by solid black arrows). This indicates that the resonance at 43.95 GHz primarily exhibits an electric nature, as the surface current vectors on the top and bottom surfaces are parallel, intensifying the electric field within the dielectric substrate.

Lastly, Fig. 8d depicts the SCD on the top and bottom surfaces of the structure at 52.1 GHz. Here, the surface vectors flow from the top-left to the bottom-right on both the ring and square’s top surface (indicated by solid black arrows). However, on the bottom metallic plate, the surface vectors flow in the opposite direction, from bottom-right to top-left (as indicated by solid black arrows). This indicates that the resonance at 52.1 GHz primarily exhibits a magnetic nature, as evidenced by the anti-parallel surface current vectors on the top and bottom surfaces, intensifying the magnetic field within the dielectric substrate.

Figure 9 illustrates the SCD at the center frequencies corresponding to the CP bands, specifically 16.32 GHz, 19.82 GHz, 32.20 GHz, and 57.7 GHz. One prominent observation across these frequency bands is the arrangement of surface currents in both the top and bottom layers, forming an orthogonal configuration. This is notably distinct from the LP scenario, where all currents in the top and bottom layers align along a particular diagonal direction. The orthogonal alignment, depicted by solid vertical arrows on the top and solid horizontal arrows on the bottom in Fig. 9, serves as an indication of the CP characteristic of the reflected wave.

**Fig. 10:**
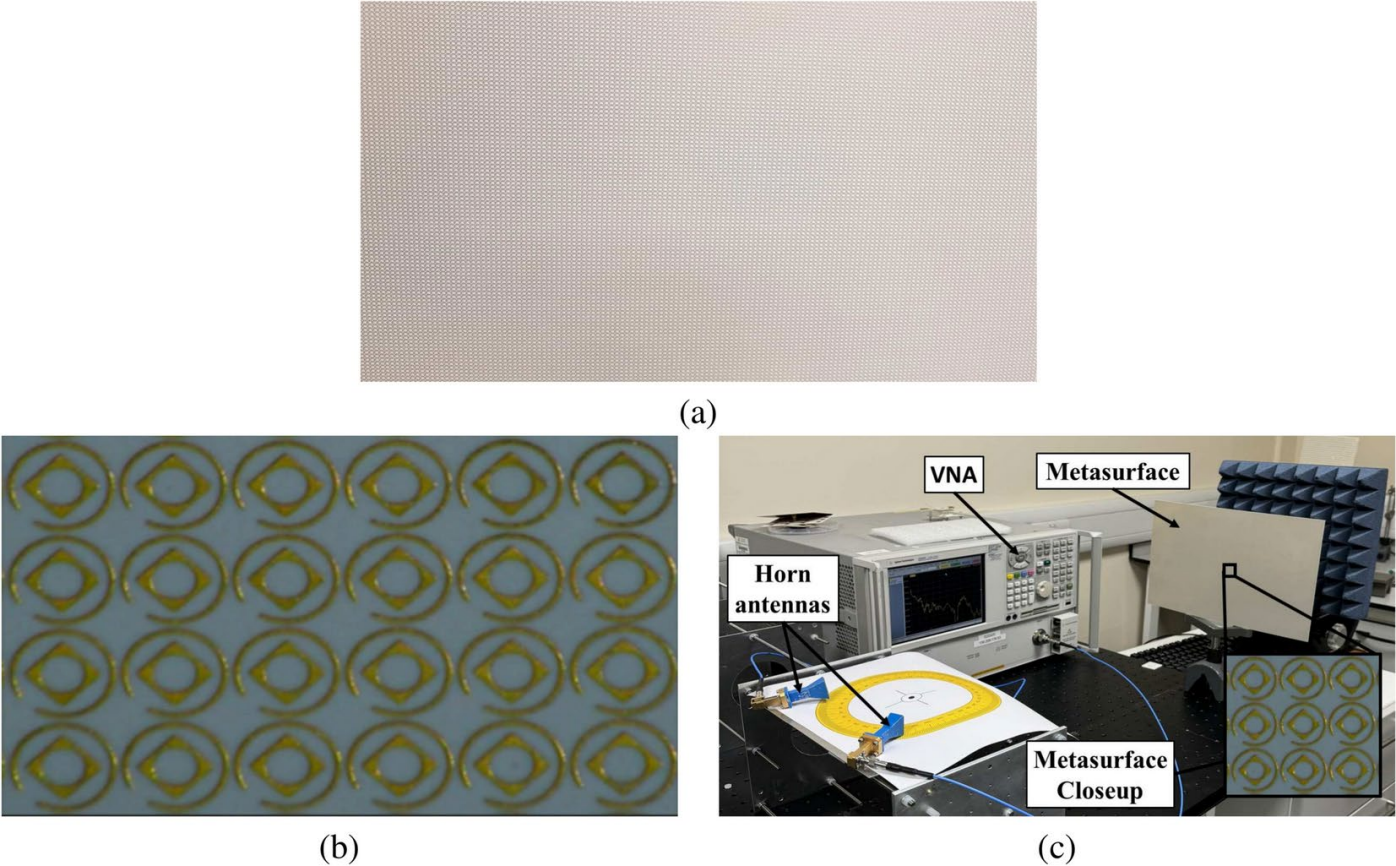
Free-space real-time experiment (**a**) full metasurface with a grid of 100 x 140 unit cells (**b**) metasurface closeup with a grid of 4 x 6 unit cells (**c**) experimental setup.

## **Fabrication and experimental measurements**

We initiated our study by simulating the co-reflection ($\text {R}_{yy}$) and cross-reflection ($\text {R}_{xy}$) coefficients of the reflective metasurface using CST Microwave $\text {Studio}^{\circledR }$. To validate the real-world performance of our designed reflective metasurface polarization converter, we proceeded with experimental measurements on the fabricated prototype. Figure 10a, b shows a full metasurface grid of $100 \times 140$ unit cells, and metasurface closeup with a grid of $4 \times 6$ unit cells, respectively. The experimental setup, shown in Fig. 10c, employed two multi-band horn antennas to illuminate the metasurface and capture the reflected electromagnetic waves. An Agilent PNA network analyzer N5224A was used for signal transmission and reception, with TRL calibration ensuring accurate transmission line calibration. To measure the co-reflection coefficients, both horn antennas were aligned vertically, whereas, for cross-reflection coefficients, one antenna was rotated by 90 degrees to achieve the necessary polarization state. This approach ensured a comprehensive validation of the metasurface’s polarization conversion capabilities across the targeted frequency bands.

**Fig. 11:**
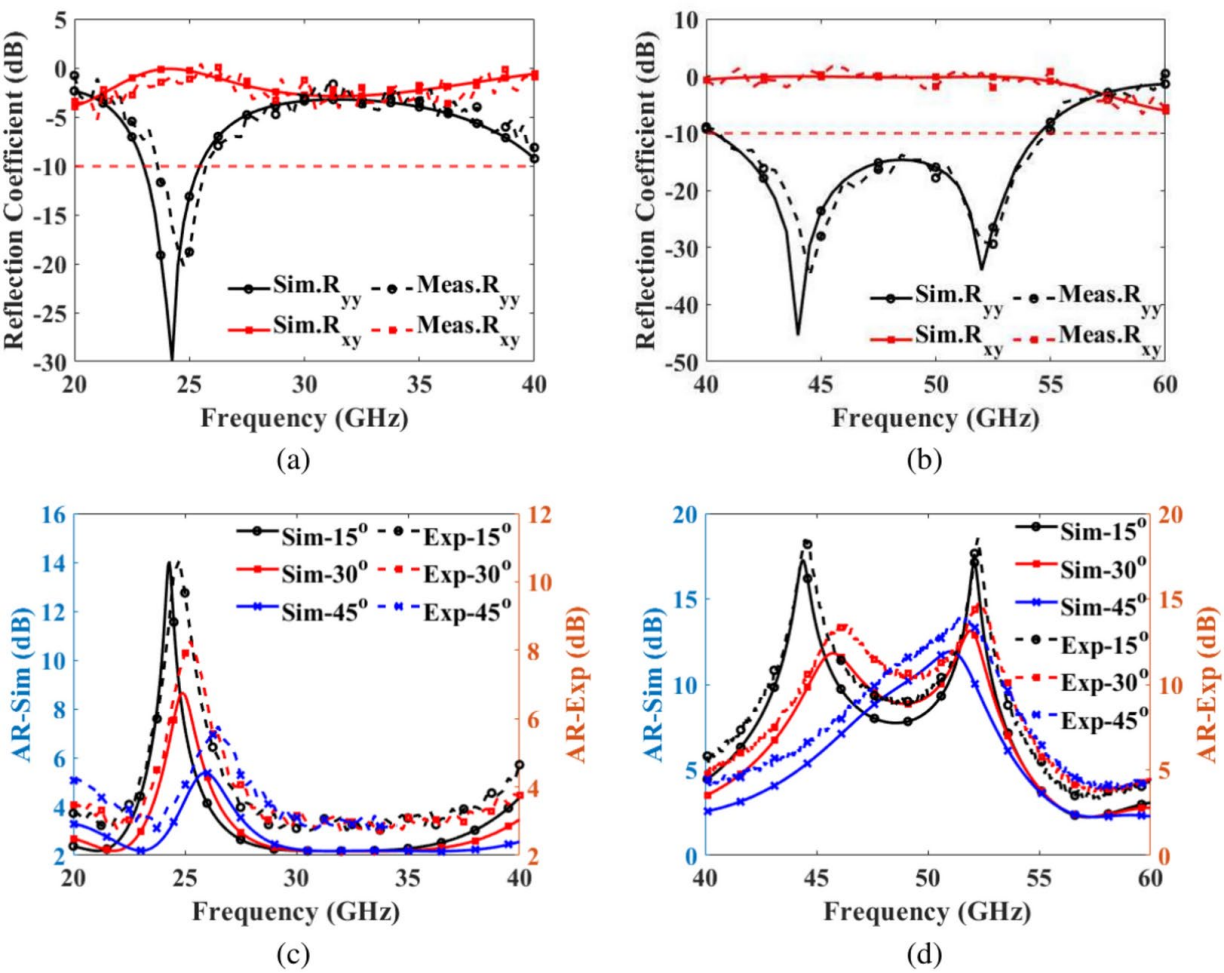
Simulation versus measured results (**a**) co- and cross-reflection coefficients for 20-40 GHz (**b**) co- and cross-reflection coefficients for 40-60 GHz (**c**) AR for 20-40 GHz (**d**) AR for 40-60 GHz.

The fabricated metasurface sample was positioned at a calculated distance from the horn antenna to ensure normal incidence of the EM waves. The network analyzer was configured to sweep across the 20-40 GHz and 40-60 GHz frequency bands, capturing reflection coefficients for both co-polarization and cross-polarization components. Co-polarization reflection was measured with aligned transmitted and received polarization orientations, while cross-polarization reflection was recorded with orthogonal orientations. The collected data offered valuable insights into the metasurface’s polarization conversion efficiency and AR, validating its performance across the targeted frequency ranges. This method effectively showcased the metasurface’s high PCR, demonstrating its potential for various practical applications.

**Table 3:** Performance comparison with state of the art.

Study	Frequency band	Operating band (GHz)	BW (GHz)	Type	FB	PCR	AS	Length (mm)	Thick (mm)
^21^	Ku	15.5-16.5 17.4-18.38 12.8-13.5	1 0.95 0.70	LP LHCP RHCP	6.25% 5.31% 5.21%	$\ge 85$%	$\le 60$%	0.28$\lambda _\circ$	0.09$\lambda _\circ$
^22^	Ku	12.0-17.9 11.3-11.5	5.9 0.2	LP LHCP	39.4% 1.75%	$\ge 80$%	$\le 45$%	0.30$\lambda _\circ$	0.05$\lambda _\circ$
^23^	X,Ku,K	12.3-17.8 10.8-11.0 21.8-27.4	5.5 0.02 5.6	LP LHCP LHCP	36.5% 1.82% 22.5%	$\ge 80$%	$\le 50$%	0.35$\lambda _\circ$	0.09$\lambda _\circ$
^24^	C,X,Ku,K	5.3-5.4 7.2-8 8.8-11.2 14.9-20.2	0.1 0.8 2.4 5.3	LP LP RHCP RHCP	1.8% 10.5% 24% 30.1%	-	$\le 75$%	0.20$\lambda _\circ$	0.09$\lambda _\circ$
^25^	Ku,Ka	11.05-16.75 34.16-43.03	5.7 8.87	LHCP LHCP	23% 41%	-	$\le 45$%	0.06$\lambda _\circ$	0.001$\lambda _\circ$
^26^	Ku,K,Ka	15-21.2 16.3-21.1 27-30.3	6.2 6.1 3.3	LHCP RHCP LHCP	34.3% 25.7% 11.5%	-	$\le 45$%	0.23$\lambda _\circ$	0.13$\lambda _\circ$
^27^	X,Ku,K,Ka	10.6-10.92 12.12-17.32 22.72-37.76 11.41-12 19.0-22.34 40.74-46.82	0.32 5.2 15.04 1.09 3.33 6.08	LP LP LP LHCP RHCP LHCP	2.97% 35.33% 49.74% 5.04% 16.10% 13.88%	$\ge 90$%	$\le 45$%	0.23$\lambda _\circ$	0.09$\lambda _\circ$
^28^	Ka,U	27.38-28.61 33.1-52	1.23 18.9	LHCP RHCP	4.3% 44.4%	-	$\le 25$%	0.59$\lambda _\circ$	0.06$\lambda _\circ$
This study	Ku,K,Ka,U	16.2-17.2 23.0-25.4 40.3-54.35 16.1-16.55 17.5-22.15 26.65-37.75 55.6-59.8	0.5 2.4 14.05 0.45 4.65 11.1 4.2	LP LP LP RHCP LHCP RHCP RHCP	2.94% 9.91% 29.6% 2.75% 23.45% 34.47% 7.27%	$\ge 90$%	$\le 45$%	0.23$\lambda _\circ$	0.08$\lambda _\circ$

To ensure accurate measurements, we maintained the far-field distance (d) between the metasurface and the Tx/Rx antennas, calculated using the criterion $2D^2/\lambda _\circ$ where D is the metasurface dimension and $\lambda _\circ$ is the free space wavelength at 14 GHz^35^. The effect of this distance on performance primarily arises from free space path loss, which is governed by the logarithmic equation $20\text {log} (4\pi d/\lambda _\circ )$. While greater distances lead to increased path loss, this does not critically impact the metasurface’s performance in polarization conversion at the desired frequency bands. Through testing, we observed a path loss reduction of less than 2 dB within a 10 cm distance variation. In practical scenarios where path loss could pose a significant issue, mitigation strategies like employing high-gain antennas or incorporating high-power amplifiers at the transmission side could be considered, though such strategies are beyond the scope of this study.

Figure 11a, b presents a comparison between experimentally obtained co-reflection ($\text {R}_{yy}$) and cross-reflection ($\text {R}_{xy}$) coefficients with corresponding simulated results across the 20-40 GHz and 40-60 GHz frequency ranges. The strong correlation between simulation and experimental data demonstrates the reliability of our design, with minor deviations likely arising from fabrication tolerances and the inherent challenges of working with small-scale prototypes. Similarly, Fig. 11c, d illustrates the experimental and simulated AR values in decibels across the same frequency ranges. Due to horn antenna limitations, measurements at normal incidence were not feasible; however, experimental data were collected at incident angles of $15^\circ$, $30^\circ$, and $45^\circ$. Across these angles, experimental and simulated results show a strong agreement, with experimental AR values closely matching simulated predictions. A slight central frequency shift is noted, likely due to minor fabrication inaccuracies, dielectric constant variances, antenna misalignment, or environmental noise during testing. Despite these small variations, the alignment of experimental and simulated data highlights the design’s robustness and precision, demonstrating its reliability across practical scenarios.

A comparative analysis was conducted with existing works^21–28^. The focus was on the operating band, polarization type (LP, CP), PCR, AS, FB, and unit cell length(mm) in relation to wavelength $\lambda _\circ$ where $\lambda _\circ =c/f_\circ$, and $f_\circ =35$ GHz.

The detailed quantitative performance analysis presented in Table 3 underscores the competitive advantages of our proposed metasurface converter. In previous works^21–28^, the unit cell lengths consistently exceed 2 mm, while only a few studies^25,28^ report unit cell thicknesses of 0.127 mm and 0.51 mm, respectively. In contrast, most designs have a thickness greater than 0.76 mm. Compared to these, our proposed metasurface features a more compact unit cell with dimensions smaller than those found in existing studies, demonstrating a significant improvement in size reduction without compromising functionality.

Maintaining over 90% PCR even at higher incidence angles (up to $45^\circ$) aligns with previous studies^27^, and outperforming work in prior research^21–26,28^. Similarly, its AS aligns with previous studies^22,25–28^, outperforming work in^28^. Moreover, our metasurface converter exhibits superior performance in terms of FB for LP compared to previous work^21–26,28^.

The proposed reflective metasurface achieves CP across four distinct frequency bands, offering 0.45 GHz bandwidth in the Ku-band (RHCP), 4.65 GHz in the K-band (LHCP), 11.10 GHz in the Ka-band (RHCP), and 4.20 GHz in the U-band (RHCP). This unique characteristic provides four distinct, non-adjacent frequency bands, which is essential for ensuring isolation between transmitted and received signals in Satcom systems. Unlike previous studies^21–28^, none have demonstrated four distinct and non-adjacent frequency bands, a critical feature for Satcom applications.

Remarkably, our proposed study showcases distinctive strengths in polarization conversion, particularly excelling in LP, LHCP, and RHCP conversion categories. LP, and CP conversion are vital in satellite communications, particularly in the Ku, K, Ka, and U bands. LP is often used for point-to-point communication links, enabling simple antenna designs with high efficiency. However, CP, including LHCP, and RHCP, offers better resistance to signal degradation due to atmospheric conditions like rain and polarization mismatch.

In the Ku and K bands, CP is commonly applied for satellite broadcasting and Earth observation missions, ensuring signal reliability and reducing interference. In the Ka band, CP supports high-data-rate communication for broadband services and deep-space missions. Meanwhile, in the U band, CP plays a critical role in advanced military applications and satellite tracking systems. The ability to switch between LP and CP within these frequency bands enhances versatility in communication systems, allowing adaptive polarization control for various satellite operations, optimizing signal strength, and improving overall system performance across different atmospheric and environmental conditions.

In summary, our asymmetric reflective metasurface surpasses existing metasurfaces, making a significant contribution to the research landscape in LP and CP conversion within the field.

## **Conclusion**

In conclusion, we have successfully designed, simulated, and experimentally validated a multi-band reflective metasurface polarization converter capable of efficiently linearly polarization and circular polarization across a wide frequency spectrum. Through a careful arrangement of ring and square elements, the metasurface achieves polarization conversion in multiple frequency bands, as evidenced by simulation and experimental results. The analysis of induced surface current distributions provides insights into the underlying mechanisms driving polarization conversion, further validating the effectiveness of the proposed design. Overall, the demonstrated performance characteristics of the metasurface, underscore its significance in advancing polarization control technology.

## Data Availability

Data sets generated during the current study are available from the corresponding authors on reasonable request.
